# Bone mineral density fall during aromatase inhibitor treatment may predict lower breast cancer recurrence

**DOI:** 10.1002/cam4.6846

**Published:** 2024-01-08

**Authors:** Hilary Martin, Andrew Redfern

**Affiliations:** ^1^ Medical Oncology Fiona Stanley Hospital Murdoch Western Australia Australia; ^2^ School of Medicine University of Western Australia Perth Western Australia Australia

**Keywords:** bone mineral density, breast cancer

## Abstract

**Purpose:**

Aromatase inhibitors (AIs) are associated with reduction in bone mineral density (BMD). The use of bone strengthening agents zoledronic acid and denosumab are associated with improved breast cancer outcomes for post‐menopausal patients treated with AIs. This study investigates whether change in BMD with AI therapy is associated with breast cancer recurrence.

**Methods:**

A cohort of patients treated at a single institution diagnosed with hormone receptor‐positive breast cancer with baseline BMD and subsequent BMD test while receiving adjuvant aromatase inhibitor therapy were studied. Demographic, treatment and outcome data was obtained. Simple and multiple linear regression analysis was performed to investigate predictors of annual percent BMD change at the LS and hip. Univariate and multivariate Cox proportional hazards modelling were undertaken to investigate predictors of breast cancer recurrence.

**Results:**

353 patients eligible patients were identified. In multivariate analysis of lumbar spine BMD change, the difference between those in quartile 1, which showed the greatest reduction in BMD, and quartile 3, with substantially less reduction, was significant (HR = 3.02, 95% CI 1.15–7.90 *p* = 0.025). Hip BMD reduction was also not significantly associated with breast cancer recurrence. The two quartiles with the least reduction in hip BMD showing a non‐significant reduced risk of recurrence relative to the quartile with the greatest (*p* = 0.10).

**Conclusions:**

The findings suggest an association may exist between lumbar spine BMD change and breast cancer recurrence for patients treated with adjuvant AI. Further research is required to determine whether BMD change can be utilised as a biomarker.

## 1 INTRODUCTION

Bone mineral density (BMD) in females is modulated by oestrogen.^1^ Falling oestrogen levels during the transition through menopausal are associated with increased bone loss, which result in a reduction in BMD.^2^, ^3^ Similarly, lowering oestrogen levels to below those usually associated with menopause by the use of aromatase inhibitors (AIs) results in further bone loss,^4^, ^5^ with greater loss of BMD in patients treated with AIs compared those treated with tamoxifen or placebo.^4^, ^5^, ^6^, ^7^, ^8^

Links between the benefit of treatment and the treatment side effects have been suggested with other anti‐neoplastics, including neutropaenia with chemotherapy,^9^ hypertension with bevacizumab^10^, ^11^ and skin rash with EGFR blockade.^12^ Hypotheses explaining such links include: the existence of common biological elements between the tissue affected and targeted neoplastic cells, higher drug exposure or activation due to individual pharmacokinetics, or that those with higher compliance experience both greater benefit and more treatment‐related toxicity. The data regarding toxicity and efficacy has been inconsistent for endocrine therapy.^13^, ^14^, ^15^, ^16^, ^17^, ^18^ Studies reported regarding endocrine treatment toxicity and outcome have focused on symptoms including arthralgias, vasomotor symptoms and genito‐urinary symptoms. No studies reported to date have investigated the association between BMD change while on endocrine therapy and breast cancer recurrence or mortality.

Considering the impact of therapy on the composition of normal tissues, reproducible correlations between fall in radiological breast density and improved outcomes on tamoxifen have been shown in a number of studies,^19^, ^20^, ^21^, ^22^ with a non‐statistically significant trend towards improved outcome shown in one study.^23^

With these precedents for the prediction of adjuvant tamoxifen benefit by reduction in breast stromal density and the links observed across a range of anti‐neoplastic treatments between toxicity and benefit we hypothesised that patients experiencing more marked BMD falls on AI therapy may experience better outcomes than those with lesser or no change. Should change in BMD be predictive of disease recurrence, such change could be utilised to guide patient management. In particular, a favourable association between higher BMD loss on AIs and outcome would dissuade discontinuation of treatment due to toxicity. As far as we are aware it is the first study to investigate this association.

## 2 METHODS

### 2.1 Patient population

A cohort of patients treated at a single institution, Royal Perth Hospital, Western Australia, diagnosed with hormone receptor‐positive breast cancer between January 1994 and December 2011 planned for treatment with curative intent were identified from the prospectively‐populated hospital breast unit database which holds clinicopathologic, surgical treatment and early outcome data. Census date for follow‐up data was 1st December 2016. Patient endocrine therapy and chemotherapy treatment data, smoking and alcohol history, body mass index (BMI), concomitant medications including bisphosphonates or denosumab, hormone replacement therapy (HRT) usage as well as additional outcome data were obtained from case notes, clinic letters and hospital information systems. Data were cross‐referenced with the Western Australian Cancer Registry to confirm mortality information.

HRT use was categorised as ‘never’ HRT was never used, ‘current use’ for those using HRT at time of diagnosis and ‘former use’ if ceased greater than 6 months from diagnosis. Menopausal status was obtained from the case notes. Women with unclear menopausal status were coded as premenopausal if <45yo, perimenopausal if 45–55 and post‐menopausal if >55, based on median age of menopause for this population.

### 2.2 BMD measurement

BMD data were obtained from standard report information held in case notes, clinic letters and the state‐based electronic radiology system reports. For inclusion in this study, patients required BMD results available from a baseline dual‐energy X‐ray absorptiometry (DEXA) scan performed up to 3 months prior to, or within 3 months of commencement of an AI, and a subsequent follow‐up DEXA scan at least 6 months after the initial scan undertaken while still on AI treatment. This timing was consistent with clinical practice for baseline and subsequent bone density measures. There was no maximum duration of AI treatment prior to the subsequent DEXA scan for inclusion.

Data regarding T scores and BMD were collected for the LS and for the hip. The proportion of patients with BMD in each category; osteoporosis, osteopenia or normal based on the *T* score were analysed at baseline and the subsequent scan. T score classifications were normal ≥−1.0, osteopenia between −2.5 and −1.0, osteoporosis ≤− 2.5. The percentage of annual change in BMD at both the lumbar spine (LS) and hip were calculated using the baseline BMD measurement minus the next sequential BMD while on treatment with an AI divided by the baseline scan BMD and then divided by the number of years between scans.

As rate of BMD change varied over time from AI initiation to subsequent BMD testing in previous studies,^5^, ^6^ data was also compared between those with subsequent BMD performed between 11 and 13 months following initial BMD to determine data for change within approximately 12 months of endocrine therapy, and those with BMD testing performed at a greater than 13 months from initial BMD. Patients known to be on a bisphosphonate or denosumab either at commencement of AI or at any time between the two DEXA scans used for analysis were excluded due to potential impact upon BMD.

### 2.3 Statistical analysis

Simple and multiple linear regression analysis was performed to investigate predictors of annual percent BMD change at the LS and hip separately. Potential predictors examined were age at time of baseline BMD, menopausal status at diagnosis, body mass index (BMI) at diagnosis, smoking status, alcohol use, HRT use, receipt of chemotherapy, whether AIs were given initially or subsequent to tamoxifen, time from baseline BMD to subsequent BMD and baseline BMD. Tests of linearity, homoscedasticity and normality were undertaken to confirm that the linearity assumption was valid for each variable.

In addition, the association between annual percent change LS BMD and hip BMD was examined. The correlation between these variables was tested using Pearson's correlation. Linear regression testing was also undertaken.

The primary outcome was new breast cancer event rate, hereafter referred to as recurrence and could include locoregional recurrence, distant recurrence or development of a contralateral breast cancer. Univariate and multivariate Cox proportional hazards modelling were undertaken to investigate predictors of recurrence. The variables of primary interest were change in BMD at the LS and change in BMD at the hip. Co‐variables examined on univariate modelling were age at diagnosis, BMI at diagnosis, menopausal status at diagnosis, tumour size, tumour grade, nodal involvement, presence of lymphovascular invasion (LVI), alcohol consumption, smoking status at diagnosis and baseline BMD at hip and LS. The associations between both LS and hip BMD annual percent changes and recurrence were non‐linear and so these variables were analysed as quartiles of change. The association between age and recurrence, as well as BMI, were also non‐linear and therefore were analysed as categorical variables.

Proportional hazards testing was undertaken. The analysis was rerun excluding those recorded to have been prescribed a bisphosphonate or denosumab following the second BMD test. Exploratory univariate Cox regression analysis was undertaken examining the associations between LS BMD change or hip BMD change, and the secondary endpoints of bone metastases development, breast cancer related mortality, and overall survival. STATA IC 14 were used for analysis.

Ethical approval was given by the Royal Perth Hospital Human Research Ethics Committee and the University of Western Australia Ethics Committee, HREC registration number 13‐014.

## 3 RESULTS

### 3.1 Patient and BMD demographics

1047 patients who received AI were identified, 947 of whom had had at least one DEXA scan available for review and 362 had at least two BMDs recorded in the required timeframe. Of this group seven who were on bisphosphonate prior to commencement of AI therapy and two who commenced bisphosphonate prior to their second DEXA scan were excluded leaving 353 patients eligible for analysis.

Baseline characteristics and treatment data are shown in Table 1. The majority (7*4.8%*) were post‐menopausal at diagnosis, and most received AI as initial endocrine therapy. Median follow‐up was 7.3 years (range 1.1 to 17.0 years). There were 45 recurrences in the analysed cohort. A total of 29 patients received subsequent bisphosphonates at some point following their second DEXA scan.

**TABLE 1:** Patient characteristics.

Characteristic	*N* (%)
Age at baseline (yrs)	
<50	34 (9.6)
50–65	199 (56.4)
65–80	104 (29.5)
>80	16 (4.5)
Menopausal status at diagnosis	
Pre‐menopausal	58 (16.4)
Peri‐menopausal	31 (8.8)
Post‐menopausal	264 (74.8)
HRT usage	
Never	239 (69.5)
At diagnosis	47 (13.3)
Previous	58 (16.4)
Unknown	9 (2.6)
Smoking history	
Never	239 (65.7)
At diagnosis	52 (14.7)
Previous	61 (17.2)
Unknown	8 (2.2)
Alcohol intake	
No	151 (42.8)
Yes	184 (52.1)
Unknown	18 (5.1)
BMI at diagnosis	
<25	103 (29.2)
25–29.9	103 (29.2)
30–34.9	83 (23.5)
>35	64 (18.1)
Tumour grade	
1	75 (21.3)
2	214 (60.6)
3	59 (16.7)
Unknown	5 (1.4)
Tumour size (cm)	
≤2.0	188 (53.3)
>2–5.0	133 (37.7)
>5.0	28 (7.9)
Unknown	4 (1.4)
Lymph node status	
0	176 (49.9)
1–3	124 (35.1)
4+	52 (14.7)
Unknown	1 (0.3)
HER2 status	
0	101 (28.6)
1	199 (56.4)
2	28 (7.9)
3	21 (6.0)
Adjuvant chemotherapy	
No	158 (44.8)
Yes	195 (55.2)
Aromatase inhibitor therapy	
Upfront treatment	
Letrozole	102 (28.9)
Anastrazole	132 (37.3)
Exemestane	5 (1.4)
AI + LHRH agonist	8 (2.2)
Switched treatment	
Letrozole	38 (11.1)
Anastrazole	59 (16.7)
Exemestane	16 (4.5)
AI + LHRH agonist	3 (0.9)

Baseline and subsequent T scores were available for LS BMD on 349 patients and for hip BMD on 342 patients. The distribution of patients by BMD T score category is shown in Table 2. Mean baseline and subsequent BMD, mean BMD change and annual BMD change are shown in Table 3.

**TABLE 2:** Baseline and follow‐up DEXA scan *T* score demographics.

	Baseline		Follow‐up	
	*N*	(%)	*N*	(%)
Lumbar spine				
Normal	213	(61.0)	173	(49.6)
Osteopenia	133	(38.1)	154	(44.1)
Osteoporosis	3	(0.9)	22	(6.3)
Total hip				
Normal	263	(76.9)	228	(66.7)
Osteopenia	78	(22.8)	112	(32.8)
Osteoporosis	1	(0.3)	2	(0.6)

**TABLE 3:** Absolute mean bone mass change across study.

	*N*	Baseline	(Range)	Follow‐up	(Range)	Change	
						Absolute	Percent
LS BMD	337	0.99	(0.72–1.50)	0.95	(0.65–1.50)	−0.037	−2.01
Hip BMD	329	0.91	(0.61–1.40)	0.87	(0.61–1.30)	0.037	−2.18

The time from baseline to subsequent study ranged from 0.59 years to 6.23 years with median of 1.85 years. Mean reduction in LS BMD was significantly greater for the 243 patients with an interval greater than 12 months than the 89 with a repeat within 12 months (4.29 vs. 2.04%, *p* = 0.001). A similarly greater hip BMD reduction was seen for the 237 patients with later compared to the 87 patients with and earlier rescans (4.69 vs. 1.92%, *p* < 0.001). In order to adjust for the differing time intervals between DEXA scans and resulting variation in BMD reduction, annual percent change BMD was calculated and utilised for further analyses. There was no significant difference by independent *t*‐test in annual percent reduction between patients with less than or more than 2.5 years between DEXA scan results for either LS (2.5 vs. 1.85%, *p* = 0.69) or hip (2.26 vs. 1.79%, *p* = 0.23).

### 3.2 Predictors of change in bone mineral density

Simple and multiple linear regression analyses were performed of candidate factors (Table 4). In both simple and multiple linear regression age at first BMD, baseline BMI, baseline LS BMD and menopausal status were significantly associated with LS BMD reduction. No significant interactions were found. Hence, increasing age at diagnosis and higher baseline BMI were associated with less reduction in LS BMD whereas higher baseline BMD and pre‐menopausal status correlated with greater reduction. In simple linear regression baseline hip BMD, smoking status, baseline BMI and age were significantly associated with change in hip BMD, and all but age remained significant in multiple linear regression. Thus, greater BMD falls occur in current smokers and those with higher baseline BMD, whereas lesser falls are associated with higher BMI. All variables met the linear regression assumptions of linearity, homoscedasticity and normality.

**TABLE 4:** Correlates with reduction in BMD.

Variable	Lumbosacral spine BMD reduction correlate						Hip BMD reduction correlate					
	Simple linear regression			Multiple linear regression			Simple linear regression			Multiple linear regression		
	Co‐efficient	95% CI	*p*	Co‐efficient	95% CI	*p*	Co‐efficient	95% CI	*p*	Co‐efficient	95% CI	*p*
Age of first BMD	0.07	0.032 to 0.11	<0.001	0.061	0.023–0.99	0.002	0.012	−0.019 to 0.042	0.45	–	–	‐–
Menopausal status			0.03						0.07			
Post‐menopausal	Reference	–	–	–	–	‐–	Reference	–	–	–	–	–
Peri‐menopausal	−0.8	−1.8 to 0.21	0.12	–	–	–	−0.97	−2.00 to 0.07	0.067	–	–	–
Pre‐menopausal	−1.58	−2.88 to −0.27	0.018	–	–	–	−0.64	−1.43 to 0.15	0.11	–	–	–
BMI at baseline	0.075	0.017 to 0.13	0.011	0.087	0.028 to 0.15	0.004	−0.064	−0.11 to −0.019	0.006	0.46	0.13 to 0.79	0.007
Current smoking												
No	Reference	–	–	–	–	–	Reference	–	–	–	–	–
Yes	0.044	−1.00 to 1.09	0.935	–	–	–	−0.86	−1.67 to −0.058	0.036	−0.98	−1.75 to 0.20	0.013
HRT use			0.38									
Never	Reference	–	–	–	–	–	Reference	–	–	–	–	–
Current	0.33	−1.47 to 0.81	0.57	–	–	–	−0.13	−1.01 to 0.75	0.77	–	–	–
Past	0.6	−0.42 to 1.61	0.25	–	–	–	0.033	−0.76 to 0.82	0.93	–	–	–
Timing of first AI												
Initial therapy	Reference	–	–	–	–	–	Reference	–	–	–	–	–
After tamoxifen	−0.71	−1.51 to 0.08	0.079	–	–	–	−0.51	−1.13 to 0.11	0.1	–	–	–
Baseline BMD	−3.36	−6.04 to −0.67	0.014	−4.58	−7.31 to −1.86	0.001	−5.37	−7.71 to −3.03	<0.001	11.31	0.06 to 22.6	0.049

Baseline hip BMD interacted significantly with baseline BMI and thus was included in the final model (*p* = 0.004). There was only weak correlation between annual percent changes of LS and hip BMD with Pearson's correlation coefficient of 0.183. In simple linear regression analysis these factors significantly associated with each other (coefficient = 0.141 95% CI 0.058 to 0.224, *p* = 0.001). When added to the final multiple regression models, annual percent change in hip BMD was also significantly associated with annual percent change in the LS and vice versa.

### 3.3 Bone mineral density change and breast cancer recurrence

The results for univariate and multivariate analyses for variables found to be significantly associated with recurrence in the best fitting Cox regression model of breast cancer recurrence are shown in Table 5.

**TABLE 5:** Cox proportional hazards regression analysis of breast cancer recurrence.

Variable N	Univariate Analysis			Multivariate Analysis		
	HR	95% CI	*p*	HR	95% CI	*p*
Age at Diagnosis			0.092	–	–	–
<50	1	Reference	–	–	–	–
50–65	0.79	0.34–1.83	0.59	–	–	–
65–80	0.87	0.31–2.41	0.79			
>80	3.93	1.01–15.30	0.049			
BMI (kg/m^2^)			0.48			
<25	1	Reference	–	–	–	–
25–30	0.73	0.31–1.73	0.48	–	–	–
30–35	1	0.45–2.24	0.99	–	–	–
>35	0.4	0.12–1.40	0.15	–	–	–
Menopausal status			0.76			
Post‐menopausal	Reference	–	–	–	–	–
Peri‐menopausal	1.4	0.57–3.48	0.46	–	–	–
Pre‐menopausal	1.03	0.46–2.33	0.99	–	–	–
Lymph node status			0.23			
0	1	Reference	–	–	–	–
1–3	1.44	0.65–3.21	0.37	–	–	–
4–9	2.48	0.9–6.82	0.079	–	–	–
>9	2.55	0.79–8.20	0.12	–	–	–
Tumour size (cm)			0.002			
=<2	1	Reference	–	1	Reference	–
>2–<5	2.82	1.28–6.19	0.01	2.23	0.98–5.08	0.055
>5	5.44	2.02–14.61	0.001	8.98	3.14–25.7	<0.001
Tumour grade			0.084			
1	1	Reference	–	1	Reference	–
2	3.01	0.90–10.03	0.073	2.89	0.86–9.72	0.087
3	4.33	1.19–15.7	0.001	4.36	1.14–16.7	0.031
Ethanol intake			0.044			
No	1	Reference	–	1	Reference	–
Yes	2.11	1.02–4.39	0.044	2.64	1.22–5.71	0.014
Baseline BMD						
LS baseline	0.75	0.07–8.54	0.81	–	–	–
Hip baseline	1.31	0.09–18.77	0.84	–	–	–
Quartile % annual BMD fall—lumbar spine			0.3			0.15
1	1	Reference	–	1	Reference	–
2	1.31	0.47–3.62	0.6	1.76	0.62–5.01	0.29
3	2.32	0.92–5.82	0.073	3.02	1.15–7.90	0.025
4	1.47	0.53–4.05	0.12	1.75	0.61–5.04	0.3
Quartile % annual BMD fall—hip			0.23			0.1
1	1	Reference	–	1	Reference	–
2	1.47	0.64–3.42	0.365	1.47	0.61–3.56	0.4
3	0.66	0.25–1.78	0.41	0.48	0.17–1.34	0.17
4	0.66	0.24–1.86	0.44	0.69	0.24–2.02	0.5

There were 305 patients with full data available for all variables in the final model with 36 recurrence events. In univariate analysis, increasing tumour size, higher tumour grade and alcohol intake at time of diagnosis were associated with greater risk of breast cancer recurrence, although only alcohol consumption was significant in multivariate analysis.

Overall there was no significant association between recurrence and quartile of change of LS BMD (*p* = 0.15). However, there was a trend towards greater risk of recurrence for those with less reduction in BMD. In multivariate analysis, the difference between quartile 1, which showed the greatest reduction in BMD, and quartile 3, with substantially less reduction, was significant (HR = 3.02, 95% CI 1.15–7.90 *p* = 0.025). In multivariate analysis excluding hip BMD change, the relationship between higher LS BMD change and improved outcome strengthened but remained non‐significant (*p* = 0.09). Hip BMD reduction was also not significantly associated with breast cancer recurrence. The two quartiles with the least reduction in hip BMD showing a non‐significant reduced risk of recurrence relative to the quartile with the greatest (*p* = 0.10).

The proportional hazards assumption was shown to hold for this model. The associations between variables and recurrence remained similar when those who received antiresorptive therapy subsequent to second BMD assessment were excluded.

### 3.4 Breast cancer‐specific mortality, bone metastases and overall survival

Exploratory univariate Cox regression analysis was undertaken examining the interaction between these event types and LS and hip BMD change (Table 6). 21 patients developed bone metastases within the group with LS BMD results available and 20 in the group with sequential hip BMD results available. There were 18 breast cancer‐related deaths in both the group with LS BMD available sequentially and the group that had sequential hip BMD available. There were 43 deaths of any cause for those with sequential LS BMD available and 41 deaths in those with sequential hip BMD available. The association between bone metastases and LS was similar to the association seen for overall recurrence with a non‐significant reduction in bone metastases associated with greater BMD fall.

**TABLE 6:** Correlation of BMD change with bone metastases, breast cancer‐related and overall mortality.

	Bone metastases			Breast cancer mortality			Overall mortality		
	HR	95% CI	*p*	HR	95% CI	*p*	HR	95% CI	*p*
LS BMD change			0.21			0.99			0.58
1	1	Reference	–	1	Reference	–	1	Reference	–
2	2.07	0.49–8.72	0.32	0.84	0.22–3.12	0.79	0.59	0.23–1.47	0.25
3	3.7	1.00–3.7	0.05	1.02	0.29–3.56	0.97	0.79	0.34–1.80	0.57
4	1.68	0.38–7.53	0.5	0.86	0.23–3.22	0.82	1.07	0.49–2.32	0.87
Hip BMD change			0.17			0.55			0.54
1	1	Reference	–	1	Reference	–	1	Reference	–
2	8.17	1.03–64.6	*p* = 0.047	1.51	0.44–5.19	0.51	1	0.45–2.25	0.99
3	4.74	0.57–39.5	*p* = 0.15	0.58	0.13–2.60	0.48	0.57	0.23–1.41	0.22
4	3.66	0.41–32.7	p = 0.24	0.89	0.22–3.55	0.86	0.72	0.30–1.75	0.47

## 4 DISCUSSION

We report on paired sequential BMD data for 353 patients treated with AIs, providing a larger data set for analysis than either ATAC or the two BIG 1–98 bone sub‐studies.^5^, ^24^, ^25^ Furthermore, this is the first study to examine BMD change in relation to breast cancer recurrence and the first to show a possible association between BMD change and recurrence, with less reduction in LS BMD potentially being associated with a higher risk of recurrence.

No correlation was observed between baseline BMD at either site and outcome. Factors associated with change in BMD on adjusted analysis for the LS were age, BMI and baseline BMD, and at the hip were smoking status, BMI and baseline hip BMD. The associations between both age and time of menopause with rate of bone loss have been well documented.^2^, ^3^, ^26^, ^27^ The finding of attenuated BMD reduction in overweight or obese patients is consistent with those of Finkelstein et al, who found that the rate of BMD reduction was inversely correlated with BMI in pre‐ and perimenopausal patients,^2^ although contrary to a second study in older post‐menopausal women where those with excess BMI lost BMD at a greater rate than normal weight participants.^28^ This indicates that the association between BMI and BMD may not be constant across age groups. Potential modulators may include the differing influences of adipose tissue oestrogen production and weight‐bearing exercise across populations. The association between BMD loss and smoking both at the hip and the LS has been previously confirmed by a meta‐analysis^29^ and reinforces the value of smoking cessation after a breast cancer diagnosis. Consistent with the findings of BIG 1–98, smoking status was predictive for BMD change in the hip but not the LS.^24^ The mechanism is likely related to the established anti‐estrogenic impact of smoking.^30^

Compared with those with the greatest reduction in BMD at the LS, quartiles with less reduction in BMD had higher rates of further breast cancer recurrence reaching statistically significance when comparing first and third quartile of BMD reduction with a hazard ratio of 3.02 (95% CI 1.15 to 7.90, *p* = 0.025). This fits with the hypothesis that change in LS BMD associates with AI efficacy, with those with the greatest reduction in BMD, quartile 1, having the greatest benefit from treatment.

Possible explanations for a lower risk of recurrence for those with greater reduction in LS BMD include; better treatment adherence and continuation, shown to link to improved breast cancer outcome,^31^, ^32^, ^33^, ^34^ favourable pharmacokinetics or a common biologic mechanism that influences both breast cancer and bone cells. The effect was not linear with the group with the least BMD loss fairing modestly better than the third quartile of LS change. This heterogeneity may relate to the relatively low event rates with only 14 and 9 recurrences in the third and fourth quartiles, respectively. It may also be explained in part by other potential confounding factors have not been adjusted for, such as exercise or vitamin D and calcium intake, that can attenuate reduction in BMD^35^, ^36^, ^37^, ^38^ and are associated with breast cancer outcome.^39^, ^40^ Exclusion of patients who started antiresorptive therapy following the second DEXA scan did not affect the association between outcome and BMD, eliminating antiresorptive therapy, shown in other studies to be associated with improve disease‐free survival for postmenopausal women,^41^, ^42^, ^43^ as the cause of the association found in this study.

Conversely, we observed that greater hip BMD loss associated with a numerically higher risk of recurrence. Hip BMD is known to be less sensitive to oestrogen deprivation and this effect may reflect the influence of factors that link with both increased breast cancer recurrence risk and a reduction in BMD such as low exercise and low vitamin D levels. A Cochrane review showed a greater protective effect of exercise on bone mass at the hip than in the spine.^44^ Low levels of vitamin D have been shown to affect both hip and LS BMD and may under certain conditions result in greater reduction in hip BMD than LS BMD.^45^ Additionally, other biologic factors may influence bone loss as well as breast cancer progression particularly the RANK ligand signalling system (40). RANKL and RANK have been shown to be expressed in breast tissue and have been shown to stimulate breast tissue proliferation,^46^ as well as to drive osteoclast differentiation and consequent bone resorption.^47^

The strengths of this study include the number of patients included and the extent of clinical and treatment data available. However, there are a number of limitations. Median duration of follow‐up is relatively short for oestrogen receptor‐positive breast cancer at 7.3 years, with low numbers of events. Also approximately two‐thirds of the potentially eligible patient cohort were not examined as did not have sequential bone mineral density test available for analysis. It is expected that many of these patients will have had imaging undertaken outside of the hospital system through their general practitioners, but the omission of these patients may have introduced selection bias. Larger patient numbers and longer follow‐up are required to confirm or refute the observed association between BMD fall and recurrence, as well as to explore effects on bone metastases and survival. Variable BMD measurement schedules may have influenced results. Additionally, data relating to exercise and vitamin D, which may influence BMD, have not been obtained. Neither of these parameters were consistently collected for the cohort and therefore have not been included in this analysis but would be important to examine in any future research. Data was also not available on use of corticosteroids between the two bone mineral density tests, which also may have influenced results. Data on prescriptions of bisphosphonates, which could drive a positive association between BMD loss and outcome, as well as endocrine therapy adherence and compliance may be incomplete. Access to prescription data through the Australian Institute of Health and Welfare has been applied and will be examined as a proxy for adherence in future analyses. The increased use of bisphosphonates as adjuvant therapy means running a prospective study examining bone mineral density change for patients not receiving bisphosphonate would not be feasible. However examination of datasets from adjuvant studies which have included assessment of bone mineral density such as BIG‐2 or ATAC could be useful to further examine the role of BMD fall and outcome.

## 5 CONCLUSION

This study is the first to examine the association between BMD change on AI therapy and outcome. The findings suggest an association may exist between LS BMD change and breast cancer recurrence. Further research into this field is warranted, however, conducting a prospective study will be difficult due to use of adjuvant antiresorptive therapy. Examination of data from breast cancer studies with bone density and outcome information, such as BIG 1–98, ATAC and ZO‐FAST, may represent the best way forward. If confirmed, BMD change could act as a biomarker allowing tailoring of adjuvant endocrine therapy.

## AUTHOR CONTRIBUTIONS

**Hilary Martin:** Conceptualization (lead); data curation (lead); formal analysis (lead); investigation (lead); methodology (lead); writing – original draft (lead); writing – review and editing (lead). **Andrew D. Redfern:** Conceptualization (supporting); methodology (supporting); supervision (lead); writing – review and editing (supporting).

## CONFLICT OF INTEREST STATEMENT

There is no conflict of interest for either author with the respect to this publication.

## ETHICAL APPROVAL

Ethical approval was given by the Royal Perth Hospital Human Research Ethics Committee and the University of Western Australia Ethics Committee, HREC registration number 13‐014. The study was conducted in accordance with ethical standards and institutional ethics requirements.

## PRECIS FOR USE IN THE TABLE OF CONTENTS

Bone density reduction is well reported for patients on aromatase inhibitor therapy. An association may exist between bone mineral density change and breast cancer recurrence for patients treated with adjuvant aromatase inhibitor.

## Data Availability

The full data that supports the findings of this study are not publicly available as it may compromise the privacy of the study subjects. Specific non‐identifying data will be made available at request in compliance with ethics.
